# Metabolomic Profiles of Oral Rinse Samples to Distinguish Severe Periodontitis Patients From Non‐Periodontitis Controls

**DOI:** 10.1111/jre.13379

**Published:** 2025-03-14

**Authors:** Madeline X. F. Kosho, Alessio Ciurli, Martin Giera, Jacques Neefjes, Bruno G. Loos

**Affiliations:** ^1^ Department of Periodontology, Academic Centre for Dentistry Amsterdam (ACTA) University of Amsterdam and Vrije Universiteit Amsterdam Amsterdam the Netherlands; ^2^ Department of Cell and Chemical Biology and Oncode Institute Leiden University Medical Center Leiden the Netherlands; ^3^ Leiden University Medical Center Center for Proteomics and Metabolomics Leiden the Netherlands

**Keywords:** LC–MS/MS, metabolic profiling, metabolomics, oral rinse fluid, periodontal disease, periodontitis, saliva

## Abstract

**Aims:**

To explore the potential of metabolomic profiles of oral rinse samples to distinguish between patients with severe periodontitis (stage III/IV) and non‐periodontitis controls. This is coupled to an analysis of differences in metabolomic profiles between individuals without periodontitis, patients with localized periodontitis, and patients with generalized periodontitis.

**Methods:**

Periodontitis patients and controls were recruited, all aged ≥ 40 years. Study participants were asked to rinse vigorously for 30 s with 10 mL phosphate buffered saline. Metabolites were identified using a semi‐targeted liquid chromatography tandem mass spectrometry (LC–MS/MS) platform.

**Results:**

In total, 38 periodontitis patients (18 localized, 20 generalized stage III/IV periodontitis patients) and 16 controls were included. Metabolomic profiles of oral rinse samples were able to distinguish patients with severe periodontitis (stage III/IV) from non‐periodontitis controls. Among various variables for the severity of periodontitis, we found that the number of sites with deep pockets (PPD) ≥ 6 mm explained best the differences in metabolomic profiles between controls and patients with severe periodontitis. Subjects with a high number of sites with PPD ≥ 6 mm were characterized by a higher level of phosphorylated nucleotides, amino acids, peptides, and dicarboxylic acids. Metabolomic profiles were also significantly different between controls vs. generalized periodontitis and between localized periodontitis vs. generalized periodontitis (*p* < 0.05).

**Conclusion:**

Our study demonstrates that simply collected oral rinse samples are suitable for LC–MS/MS based metabolomic analysis. We show that a metabolomic profile with a substantial number of metabolites can distinguish severe periodontitis patients from non‐periodontitis controls. These observations can be a basis for further studies into screening to identify subjects with the risk of having severe periodontitis.

## Graphical abstract

Metabolomic fingerprints analyzed from oral rinses are able to distinguish between severe periodontitis (stage III/IV) patients and non‐periodontitis controls.

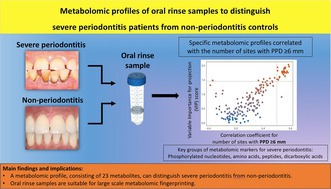

**Summary**

- Background
  ○ Promising results have been reported for the analysis of metabolomic markers in whole saliva to detect periodontitis.
  ○ Regarding the feasibility, the collection of whole saliva can be quite challenging and time‐consuming.
  ○ A possible alternative for whole saliva is the collection of an oral rinse. Currently, there is scarce evidence about metabolomic analysis in oral rinses.
- Added value of this study
  ○ The current pilot study shows that a substantial number of metabolites in oral rinse samples can distinguish severe periodontitis patients from non‐periodontitis controls.
  ○ Our study also demonstrates that simply collected oral rinse samples are suitable for identification of metabolomic markers that can detect severe periodontitis.
- Clinical implications
  ○ A series of metabolomic biomarkers for severe periodontitis found in oral rinse samples shows great potential, and this can be a basis for further studies to identify subjects with the risk of having severe periodontitis.
  ○ An oral rinse sample shows many benefits: it takes 30 seconds and it is easy to perform.
  ○ It could be a suitable collection method at physicians' offices, large epidemiological cohort studies and it has the potential for self‐testing programs.
  ○ Also, it could be convenient for patients with hyposalivation.

## 1 Introduction

Periodontitis is a chronic multifactorial inflammatory disease of the supporting structures of the teeth [1, 2]. The worldwide prevalence of all forms of periodontitis is estimated to be 62%, while the age‐standardized prevalence of severe periodontitis is 12.5% [3, 4]. Symptoms of periodontitis and its progression are often not recognized. Detection of periodontitis can occur during a dental check‐up. However, people with periodontitis, who do not regularly visit a dental practice, may experience periodontal deterioration with complaints in due time, such as periodontal abscesses and loose and exfoliating teeth. These symptoms start to become debilitating and affect quality of life [5]. Thus, a simple, non‐invasive, and easy‐to‐use method for detecting periodontitis, even with the potential for self‐testing, can be highly beneficial for individuals who do not visit the dentist for regular check‐ups.

Saliva is known to be rich in biological information and has been studied as a diagnostic fluid for oral and general health. Saliva sampling is non‐invasive, painless, and easy to perform [6]. An upcoming field in salivary diagnostics is the analysis of metabolomic profiles, which encompasses the total composition of multiple metabolites measured in this biofluid [7]. Studies have shown that oral and systemic diseases can alter the metabolomic profile, making salivary metabolites interesting potential biomarkers to detect or monitor these diseases, including periodontitis, but also diabetes mellitus (DM), Alzheimer's disease, Sjögren's syndrome (SjS), and dental caries [8, 9, 10]. Regarding periodontitis, a systematic review including 12 studies showed the potential of whole saliva to analyze metabolomic profiles for detecting and monitoring periodontitis [9].

Nonetheless, although whole saliva shows many benefits as a diagnostic fluid, the collection of whole saliva can be quite challenging for patients with oral dryness due to health conditions (such as DM and SjS) or as the result of side effects of certain medications [11, 12]. Another disadvantage of collecting whole saliva for large cohort studies (e.g., epidemiological studies) is its time‐consuming procedure [13] and the sampling at different times of the day. Especially in large cohort studies, 3–5 min of drooling to collect whole saliva would be prohibitive. A possible alternative for saliva collection is an oral rinse. An oral rinse is a suitable collection method for patients with hyposalivation and can be obtained in less time (30 s), making it also more accessible for large cohort studies. One study with 10 healthy participants showed that mouth‐rinsed water contains comparable metabolomic information as samples from collected stimulated saliva and unstimulated saliva [13]. However, the literature is scarce regarding the use of oral rinses for metabolomic analysis. Only one study described the feasibility of untargeted metabolomic analysis of oral rinses in periodontitis patients and controls. In this study, oral rinse samples were analyzed using proton nuclear magnetic resonance (^1^H‐NMR). A limited number of metabolites (3 identified and 2 unidentified) were significantly different between periodontitis patients and controls [14].

To further investigate the potential of metabolomics analysis and its application in oral health, we investigated the feasibility of using oral rinse samples to obtain comprehensive metabolomic signatures and their potential to differentiate severe periodontitis (stage III/IV) from non‐periodontitis. As a higher sensitivity alternative to NMR, we here apply a recently developed semi‐targeted LC–MS/MS platform dedicated to saliva analysis [15, 16, 17]. We hypothesized that using LC–MS/MS to analyze oral rinse samples for subsequent metabolomic analysis would provide adequate feasibility to distinguish severe periodontitis from controls. Furthermore, we aimed to investigate differences in metabolomic profiles between controls, patients with localized stage III/IV periodontitis, and patients with generalized stage III/IV periodontitis.

## 2 Methods

### 2.1 Study Design and Recruitment

All participants from this study, consisting of patients with severe periodontitis and controls, are a subset from the study population of our former cross‐sectional study, where we investigated the oral and systemic condition in periodontitis patients and controls, registered at ClinicalTrials.gov Identifier NCT03459638 and approved by the Medical Ethical Committee of the Amsterdam University Medical Center (2017.490 (A2019.151)‐NL62337.029.17). In our former study, we included consecutive patients with periodontitis that were referred to the Department of Periodontology of the Academic Centre for Dentistry of Amsterdam (ACTA) for diagnosis and treatment of periodontitis. Important to note, the individuals for the current study were included during the last several months of the enrollment period; only at the last part of the recruitment period, also oral rinse samples were obtained from the included individuals. Periodontitis patients from the former study had all stage III/IV severity and were classified as grade B or C. Therefore, this subset consists only of severe periodontitis patients. Further, individuals without periodontitis attending the ACTA clinic for general dentistry, such as for dental check‐ups or restorative procedures, were consecutively recruited as controls. There were no exclusion criteria except age: individuals were not included if their age was < 40 years. The enrollment period for all study participants was from March 2018 until March 2020. An abrupt end of recruitment was forced due to the COVID‐19 pandemic, and all clinical research activities were to be discontinued. Participants received verbal and written information about the purpose of the study and confirmed their consent. The findings were reported according to the STROBE guidelines [18].

During the first referral visit, periodontitis patients underwent a full‐mouth periodontal examination performed by periodontists or residents of the Department of Periodontology. Measurements of probing pocket depth (PPD), gingival recessions, and clinical attachment loss (CAL) were carried out for 6 sites per tooth using a manual probe. We also assessed molar furcation involvement and tooth mobility. Dental radiographs (≤ 1 year old) were used to analyze interproximal alveolar bone levels. The patients were initially screened for periodontitis according to the criteria of the Centers for Disease Control and Prevention–American Academy of Periodontology (CDC‐AAP) case definition. When positively diagnosed with periodontitis (≥ 2 interproximal sites with CAL ≥ 3 mm and ≥ 2 interproximal sites with PPD ≥ 4 mm, not on the same tooth, or one site with PPD ≥ 5 mm), subjects were asked to participate [19]. Subsequently, we applied staging (I‐IV), grading (A, B, C), and determination of the extent (localized or generalized) per stage for each periodontitis case [20].

Control subjects were included when they [1] did not fulfill the criteria for the case definition of periodontitis, [2] had not previously been treated for periodontitis, and [3] did not have interproximal alveolar bone loss on recent bitewing radiographs (≤ 1 year old); a distance of ≤ 3 mm between the cemento‐enamel junction and the most coronal part of the radiographic alveolar crest was accepted for a non‐periodontitis control subject.

### 2.2 Clinical Procedures

As this study was a subset of a larger study, a more detailed description of clinical procedures can be accessed in a previous report [21].

### 2.3 Oral Rinse Sample Collection

Oral rinse samples used in this study were collected in a subset of participants from our cohort containing periodontitis patients and controls [21]. Patients were not allowed to eat or brush their teeth for at least 1 h prior to oral rinse collection. They were instructed to swallow once before the start of rinsing with sterile Dulbecco's phosphate buffered saline (PBS, ThermoFisher Scientific, Waltham, MA, USA). The 10 mL PBS was supplied in a 50 mL centrifugation tube. Patients were asked to rinse vigorously through all sides (left, right, upper, lower) of the oral cavity for 30 s. After rinsing, patients expectorated in a 30 mL medicine cup, and consequently, the oral rinse was poured into a 50 mL centrifugation tube. Immediately after collection, the oral rinse samples were cooled on ice. Subsequently, the oral rinse samples were vortexed for 10 s, divided into aliquots of 1.0 mL in 1.5 mL screw cap microtubes, and stored at −80°C.

### 2.4 Sample Preparation

After overnight thawing at 4°C, an aliquot of 200 μL from each sample was transferred to a 1.5 mL Eppendorf tube, and 800 μL methanol (MeOH) was added. Subsequently, samples were placed in a freezer at −20°C for 20 min to complete protein precipitation. Next, samples were centrifuged at 18000 × g for 20 min at 4°C, and supernatants were transferred to 1.5 mL Eppendorf tubes. Samples were dried under a gentle stream of nitrogen and reconstituted in 40 μL of 1:99 MeOH:H_2_O (v/v %). Reconstitution was assisted by sonication for 1 min and vortexing for 5 s. For analysis of the samples, electrospray ionization (ESI) was utilized. The prepared samples were analyzed in both positive ion mode (ESI+) and negative ion mode (ESI‐) [15, 16].

### 2.5 Instrumental Parameters

Metabolites were identified using semi‐targeted liquid chromatography–tandem mass spectrometry (LC–MS/MS). Chromatography was performed using a Nexera X2 system (Shimadzu, Duisburg, Germany) equipped with a Synergi Hydro‐RP LC column (100 Å, 1.7 μm, 2 mm × 100 mm) (Phenomenex, Aschaffenburg, Germany) that was kept at 40°C. The injection volume was 10 μL, and gradient elution was accomplished using H_2_O with 0.1% formic acid (eluent A), and MeOH with 0.1% formic acid (eluent B). The flow rate was 0.4 mL min^−1^. The gradient was as follows: 0 to 1.5 min—0% B, linearly increased to 97% B at 9.9 min, 9.9 to 12.9 min—97% B, and 13.0 to 13.8 min—0% B. For detection, a Sciex TripleTOF 6600 Q‐TOF mass spectrometer (Sciex, Framingham, MS, USA) was used, scanning from mass‐to‐charge (*m/z*) 75 to 650. A detailed listing of all MS settings required to ensure reproducible MS measurements is reported in Table S1.

### 2.6 Quality Control and Batch Structure

For quality control (QC) purposes, blank samples (water) and QC pool samples (pool of all study samples) were included in the analysis. The batch structure started with an equilibration sequence of eight injections, comprising four blanks and four QC pools. Subsequently, the batch structure was as follows: one QC pool, five samples, one QC pool, and one blank until all samples had been analyzed. All samples were randomized before analysis. Instrument calibration was performed with intervals of eight injections using an integrated calibrant delivery system (Sciex).

### 2.7 Data Preprocessing

Spectra deconvolution, peak alignment, and compound identification were performed using MS‐(Data Independent AnaLysis) DIAL version 4.90, an open‐source software for identification and quantification of small molecules obtained by MS deconvolution. Based on data‐independent acquisition (DIA), molecular structures are determined in which all ions within a selected *m/z* range are fragmented and analyzed [22]. A detailed description of all MS‐DIAL settings used for detection, deconvolution, alignment, and identification is reported in Table S2. Metabolite identification was achieved by matching exact precursor mass, retention time (RT), adduct formation, and fragmentation spectra with an in‐house LC–MS/MS spectral library of authentic compounds analyzed under identical experimental conditions [17]. Based on consensus within the metabolomic field, metabolites were reported as validated (level one) when features were matched for exact mass, retention time, and MS/MS spectra using our in‐house metabolite library [23, 24]. Metabolites were reported as tentative (level three) when the library match was achieved only for exact mass and RT but without MS/MS spectra matching. Identification confidence levels are reported per metabolite in Table S3. Batch correction was performed using Locally Estimated Scatterplot Smoothing (LOESS) correction [25], which adjusts for batch effects by fitting a locally weighted regression model for each metabolite across batches, using QC samples as references to standardize each batch to the QC median response. The implemented R script for LOESS‐based correction can be found in the Rcpm package (https://github.com/RicoDerks/Rcpm) [26]. Data filtering involved: 1. the exclusion of unknown features, 2. missing value filtering by removing metabolites with signals lower than the blank average in more than 2/3 of the samples, and 3. relative standard deviation (RSD) filtering by removing any compound with an RSD higher than 30% as obtained for QC pool samples. Subsequently, all adducts belonging to the same metabolite were summed up. For metabolites identified in both ionization modes, the ion form with the higher RSD was excluded.

### 2.8 Statistical Analysis

No power calculation was performed for the current study. Therefore, the results are considered preliminary and explorative. Means, standard deviations, ranges, and frequency distributions were calculated. Demographic data and clinical parameters were compared with independent samples T‐tests or by chi‐squared tests. ANOVA or chi‐squared tests (linear by linear) were used when comparing three groups (non‐periodontitis controls, patients with localized periodontitis, and patients with generalized periodontitis). When assumptions for the parametric test were not met, we performed the non‐parametric Mann–Whitney *U* and Kruskal‐Wallis tests. The significance level was set at *p* < 0.05.

Variables related to periodontitis (extent, number of teeth with ≥ 33% bone loss, number of sites with PPD ≥ 6 mm) were analyzed by Partial Least Squares (PLS) analysis. Subsequently, a comparison of PLS models was carried out to identify the most relevant parameter. PLS analysis was followed by orthogonal PLS (O‐PLS) analysis, which was performed using the most robust PLS model, ‘number of sites with PPD ≥ 6 mm’, to further refine our variable selection process by isolating predictive components while removing orthogonal noise. Predictive Variable Importance for Projection (VIP pred) scores obtained from the O‐PLS model were cross‐analyzed with Spearman's rank coefficient of correlation to select the most promising periodontitis biomarkers. Both PLS and O‐PLS models were validated through a permutation test (permutation count of 200), model parameters (R2Y and Q2), observational diagnostics, and comparison with the score plot obtained with an unsupervised PCA; see (Figure S1–S4). Due to the nature of the variable selected in the PLS model comparison (number of sites with PPD ≥ 6 mm, a numerical variable), no further validation was conducted. However, in the case of categorical variables (e.g., extent of periodontitis), evaluating multiclass bias is recommended. Prior to multivariate data analysis computations (PCA, PLS, and O‐PLS), data were Z‐score normalized and log‐transformed.

The metabolomic dataset was analyzed using a Pairwise Wilcoxon rank sum test, and fold changes were calculated for the extent of periodontitis (control vs. localized, control vs. generalized, and localized vs. generalized periodontitis). Due to the multiple statistical tests being performed on the dataset, *p* values were adjusted using the Benjamini‐Hochberg correction (BH). Volcano plots were used to visualize and identify significant metabolites (BH‐adjusted *p* < 0.05 and fold changes > 2).

Data analyses and visualizations were carried out using SPSS 25.9.6.0.0 (IBM SPSS, Chicago, IL, USA) and R (R version 4.3.0) [27]. The packages for R used during data analysis were Tidyverse [28], ggforce [29], Scales [30], Patchwork [31], ropls [32], pheatmap [33], ggVennDiagram [34], and corrplot [35], ggpubr [36], and rstatix [37].

## 3 Results

### 3.1 Demographic Characteristics

Demographic characteristics are shown in Table 1. Patients with periodontitis, who were referred to the periodontal department and enrolled in the study, were all classified with stage III/IV periodontitis. This has resulted in the following study groups with 16 non‐periodontitis controls, 18 patients with localized stage III/IV periodontitis, and 20 patients with generalized stage III/IV periodontitis. The mean age for control subjects was 59.3 years, and for periodontitis patients, it was 54.2 years (*p =* 0.100). Among periodontitis patients, there were more current smokers (44.7%) than among controls (0.0%) (*p* = 0.002). No difference between the three groups was found for BMI (*p* = 0.354) or obese participants with a BMI ≥ 30 kg/m^2^ (*p* = 0.069). However, the total periodontitis group showed significantly more patients with obesity (26.3%) than the control group (0.0%) (*p* = 0.023). The mean HbA1c value in the total periodontitis group (5.5 ± 0.8) was not significantly different from the control group (5.1 ± 0.3 mmol/mol) (*p* = 0.140). No difference was observed between the three groups for the number of patients with self‐reported diabetes (*p* = 0.177).

**TABLE 1:** Demographic and clinical characteristics of the study population.

	Control (*n* = 16)	Localized Stage III/IV periodontitis (*n* = 18)	Generalized Stage III/IV periodontitis (*n* = 20)	*p* value^a^	Total Stage III/IV periodontitis (*n* = 38)	*p* value^b^
Age (years)	59.3 ± 10.5	51.9 ± 8.4	56.2 ± 11.2	0.113	54.2 ± 10.1	0.100
Sex						
Male	8 (50.0)	10 (55.6)	15 (75.0)	0.261	25 (65.8)	0.277
Female	8 (50.0)	8 (44.4)	5 (25.0)		13 (34.2)	
Education^**c**^						
Primary	2 (12.5)	1 (5.6)	3 (15.0)	0.587	4 (10.5)	0.391
Secondary	4 (25.0)	8 (44.4)	9 (45.0)		17 (44.7)	
>Secondary	10 (62.5)	9 (50.0)	8 (40.0)		17 (44.7)	
Smoking status						
Current	0 (0.0)	6 (33.3)	11 (55.0)	0.003	17 (44.7)	0.002
Former	6 (37.5)	6 (33.3)	7 (35.0)		13 (34.2)	
Never	10 (62.5)	6 (33.3)	2 (10.0)		8 (21.1)	
BMI (kg/m^2^)	25.4 ± 3.1	27.0 ± 3.6	27.3 ± 4.5	0.354	27.1 ± 4.1	0.150
BMI ≥ 30 (kg/m^2^)	0 (0.0)	5 (27.8)	5 (25.0)	0.069	10 (26.3)	0.023
Self‐reported diabetes	0 (0.0)	1 (5.6)	3 (15.0)	0.087	4 (7.4)	0.177
HbA1c (%)^**d**^	5.1 ± 0.3	5.3 ± 0.7	5.7 ± 1.0	0.164^**e**^	5.5 ± 0.8	0.140^**e**^
(mmol/mol)	(32.2 ± 3.1)	(34.7 ± 7.4)	(38.6 ± 10.4)		(36.8 ± 9.3)	

*Note:* Data are presented as the mean ± SD or as *n* (%).

Abbreviations: BMI, body mass index; HbA1c, glycated hemoglobin.

^**a**^ Overall *p* value for control, localized stage III/IV periodontitis, and generalized stage III/IV periodontitis.

^**b**^ Overall *p* value for control and total stage III/IV periodontitis.

^**c**^ Primary, primary education or preparatory secondary vocational education; Secondary, higher secondary general education or pre‐university education; >Secondary, beyond secondary education.

^d^ Missing values for: Localized periodontitis: *n* = 1.

^e^ Mann–Whitney and Kruskal‐Wallis tests were applied due to non‐normally distributed data.

### 3.2 Dental and Further Periodontal Parameters

Dental and further periodontal parameters are shown in Table 2. There was a significant difference between the number of teeth in generalized stage III/IV periodontitis patients (23.9), localized stage III/IV periodontitis patients (26.5), and controls (26.6) (*p* = 0.040). The rate of progression of periodontitis was classified into Grade B (28.9%) or Grade C (71.1%).

**TABLE 2:** Dental and periodontal parameters.

	Control (*n* = 16)	Localized Stage III/IV periodontitis (*n* = 18)	Generalized Stage III/IV periodontitis (*n* = 20)	*p* value^a^	Total Stage III/IV periodontitis (*n* = 38)	*p* value^b^
# Teeth	26.6 ± 2.3	26.5 ± 2.0	23.9 ± 3.9	0.040^**c**^	25.11 ± 3.4	0.216^**c**^
# Teeth with ≥ 33% bone loss	0.0	4.1 ± 2.9	14.3 ± 3.8	< 0.001	9.5 ± 6.2	NA
# Teeth with PPD ≥ 6 mm	0.0	7.3 ± 4.8	12.8 ± 7.3	< 0.001^**c**^	10.2 ± 6.8	NA
# Sites with PPD ≥ 6 mm	0.0	15.2 ± 12.5	31.6 ± 23.3	< 0.001^**c**^	23.8 ± 20.5	NA
Full mouth plaque score^**d**^	NA	53.3 ± 22.9	61.3 ± 28.6	0.357	57.4 ± 25.9	NA
Full mouth bleeding score^**d**^	NA	48.8 ± 25.9	66.1 ± 26.2	0.052	57.6 ± 27.1	NA
Grade^**e**^						
A	0.0	0 (0.0)	0 (0.0)	< 0.001	0 (0.0)	NA
B	0.0	7 (38.9)	4 (20.0)		11 (28.9)	
C	0.0	11 (61.1)	16 (80.0)		27 (71.1)	

*Note:* Data are presented as the mean ± SD or as *n* (%).

Abbreviations: #, number; NA, Not Applicable; PPD, Probing pocket depth.

^**a**^ Overall *p*‐value for control, localized stage III/IV periodontitis, and generalized stage III/IV periodontitis, except for Full mouth plaque and bleeding score: p‐value between localized and generalized periodontitis.

^b^ Overall *p* value for control and total stage III/IV periodontitis.

^**c**^ Mann–Whitney and Kruskal‐Wallis tests were applied due to non‐normally distributed data.

^**d**^ Missing values for: Localized periodontitis: *n* = 1.

^**e**^ According to World Workshop 2017 classification [20].

### 3.3 Oral Metabolome

**FIGURE 1:**
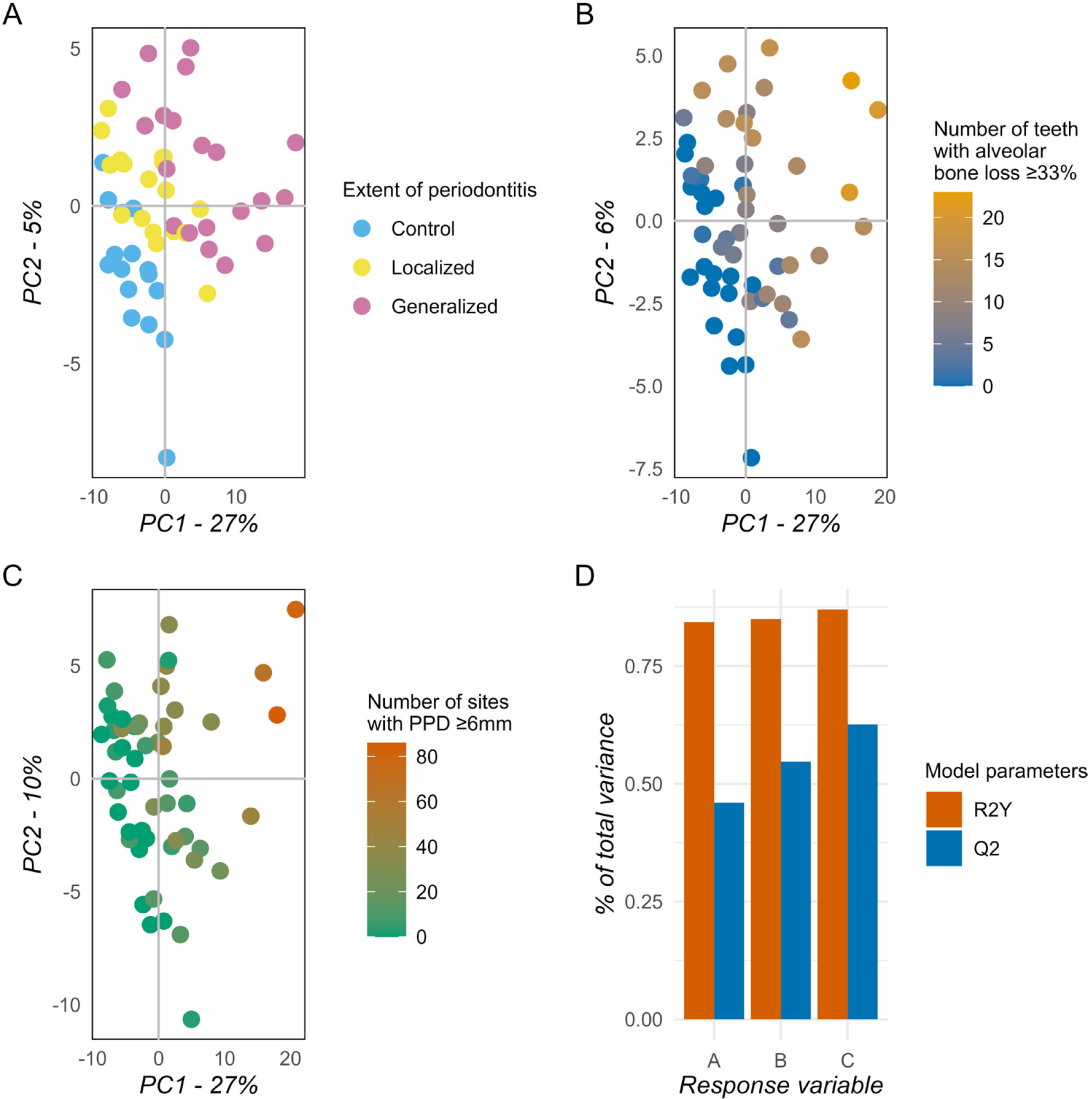
PLS model comparison. Score plots display PC1 on the x‐axis and PC2 on the y‐axis. Prior to PLS model computation, data were log‐transformed and z‐score normalized. (A) Score plot from the extent of the periodontitis‐PLS model. (B) Score plot from the number of teeth with alveolar bone loss ≥ 33%‐PLS model. (C) Score plot from the PPD ≥ 6 mm‐PLS model. (D) Model parameters comparison of A (extent of periodontitis), B (number of teeth with alveolar bone loss ≥ 33%), and C (number of sites with PPD ≥ 6 mm) by the goodness of fit (R2Y) and the predictive ability of the model (Q2) displayed in percentage of total variance (*y*‐axis). Abbreviations: PC, principal component; PPD, Periodontal Pocket Depth.

Oral rinse samples from the current cohort of 54 subjects were analyzed using a semi‐targeted LC–MS/MS platform. Parameters associated with periodontitis, including the extent (control, localized, or generalized), the number of teeth with ≥ 33% alveolar bone loss, and the number of periodontal sites with PPD ≥ 6 mm (Figure 1A–C), were analyzed through PLS analysis. PLS model comparison among the three models, generated using these periodontal parameters, identified the number of sites with PPD ≥ 6 mm as the clinical variable that best explains the oral metabolomic signature associated with periodontitis. Among the three variables, the number of periodontal sites with PPD ≥ 6 mm displayed the most robust performance in the PLS model. This was evident both qualitatively, by visual inspection of the score plot, where it exhibited the most consistent gradient across samples, and qualitatively, with the highest goodness of fit (R2Y value) and predictive power (Q2 value) (Figure 1D).

**FIGURE 2:**
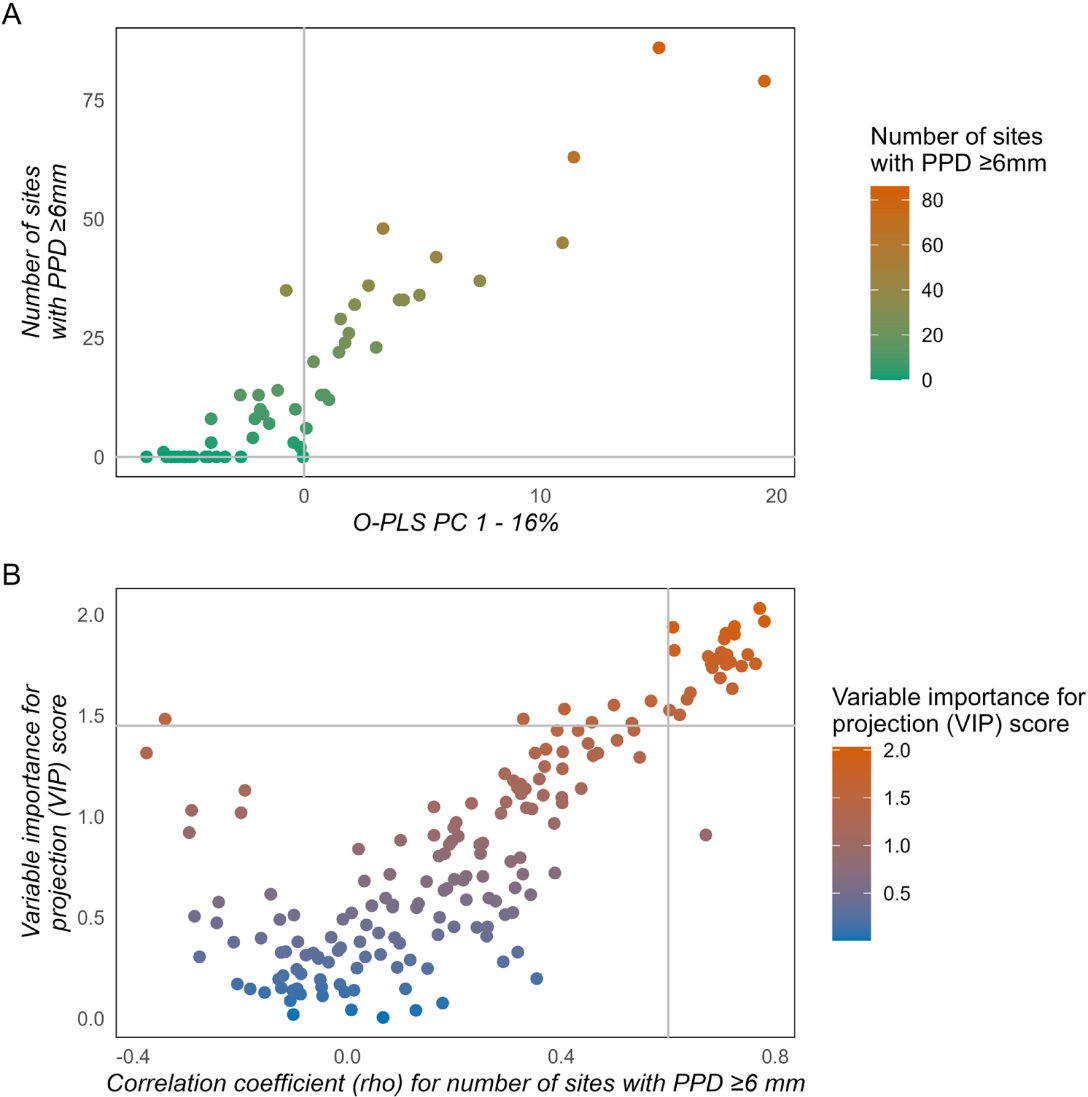
Metabolites selection for periodontitis biomarkers. (A) A scatter plot displaying the correlation between the number of sites with PPD ≥ 6 mm and PC 1 of the O‐PLS model. (B) V‐plots generated for the O‐PLS derived predictive VIP scores versus the Spearman correlation coefficients for number of sites with PPD ≥ 6 mm. Gray lines correspond to the cut‐offs used to select the most promising biomarkers, the vertical line as the correlation coefficient cut‐off, and the horizontal line as the predictive VIP cut‐off (VIP pred > 1.45 and ρ > 0.60). Abbreviations, see Figure 1, and O‐PLS, Orthogonal Partial Least Squares.

Metabolite selections as potential biomarkers for periodontitis are depicted in Figure 2, where Figure 2A shows a scatter plot displaying the correlation between the number of sites with PPD ≥ 6 mm and PC 1 of the O‐PLS model. The number of sites with PPD ≥ 6 mm was additionally analyzed using O‐PLS cross‐analyzed with Spearman's rank coefficient of correlation (ρ). A cluster of promising biomarkers was identified in the top‐right corner shown in Figure 2B (VIP pred > 1.45 and ρ >0.60). In total, 175 metabolites were identified, of which 103 are confirmed structures (level one) and 72 are tentative candidates (level three) (Table S3). Table 3, which is based on Figure 2B, shows the top candidate metabolites for stage III/IV periodontitis, in which 10 metabolites were identified as tentative. Subjects with a high number of sites with PPD ≥ 6 mm were characterized by a higher level of phosphorylated nucleotides (deoxyadenosine monophosphate, adenosine diphosphate ribose, uridine monophosphate, cytidine monophosphate, citicoline, and inosine monophosphate), amino acids (methionine, isoleucine, taurine, and norleucine), modified amino acids (diaminopimelate and O‐acetylserine), peptides (carnosine and glutathione reduced), dicarboxylic acids (3‐dehydroshikimate, methylmalonate, succinate, and malate), an amino sugar (2‐acetamido‐2‐deoxy‐beta‐D‐glucosylamine), a monosaccharide (sorbose), a polyamine (N1‐acetylspermine), an enzyme cofactor (N‐acetylglutamate), and a nucleotide sugar (uridine diphosphate‐N‐acetylglucosamine).

**TABLE 3:** Top candidate metabolites for stage III/IV periodontitis^**a**^.

Metabolites	ID level	VIP Pred	ρ	Corrected *p* value	FC Ctrl. vs. Gen.	*p* value Ctrl. vs. Gen.	FC Loc. vs. Gen.	*p* value Loc. vs. Gen.	FC Ctrl. vs. Loc.	*p* value Ctrl. vs. Loc.
2‐Acetamido‐2‐deoxy‐beta‐D‐glucosylamine	3	2.03	0.77	1.48E‐08	14.92	0.003	5.16	0.105	2.89	0.647
Deoxyadenosine monophosphate	1	1.97	0.78	7.89E‐09	7.37	0.018	4.88	0.110	1.51	0.906
Taurine^**b**^	1	1.94	0.72	2.80E‐07	3.29	0.001	2.30	0.036	1.43	0.647
Adenosine diphosphate ribose	1	1.94	0.61	4.99E‐05	10.43	0.004	4.67	0.105	2.23	0.647
Diaminopimelate	3	1.92	0.71	6.73E‐07	10.67	0.002	3.73	0.094	2.86	0.815
Uridine monophosphate	1	1.90	0.72	2.81E‐07	3.33	0.017	2.14	0.109	1.56	0.906
3‐Dehydroshikimate	3	1.88	0.70	7.84E‐07	3.81	0.003	2.22	0.071	1.71	0.647
Methionine	1	1.82	0.61	4.56E‐05	11.15	0.003	5.17	0.088	2.12	0.869
Isoleucine	1	1.81	0.70	1.05E‐06	5.64	0.003	3.40	0.036	1.66	0.906
Citicoline	3	1.80	0.75	6.46E‐08	3.49	0.015	2.09	0.178	1.67	0.900
Carnosine	3	1.80	0.71	5.93E‐07	6.05	0.002	2.99	0.036	2.02	0.937
Taurine^**b**^	3	1.79	0.67	3.40E‐06	3.50	0.005	2.49	0.082	1.41	0.900
Norleucine^**b**^	1	1.78	0.69	1.54E‐06	4.06	0.004	2.87	0.036	1.41	1
Cytidine monophosphate	3	1.76	0.72	4.25E‐07	3.23	0.003	2.35	0.036	1.37	0.906
O‐acetylserine	3	1.76	0.76	2.52E‐08	2.27	0.004	2.13	0.036	1.06	1
Inosine monophosphate	1	1.76	0.68	2.58E‐06	5.65	0.011	1.70	0.288	3.32	0.668
Gluthathione reduced	1	1.75	0.71	6.56E‐07	3.88	0.045	2.65	0.135	1.47	0.937
Sorbose	1	1.74	0.73	1.30E‐07	2.22	0.008	1.60	0.306	1.39	0.647
Norleucine^**b**^	3	1.74	0.68	2.36E‐06	3.15	0.008	2.31	0.036	1.36	0.949
Methylmalonate	1	1.69	0.70	1.16E‐06	2.19	0.016	1.84	0.036	1.19	1
Succinate	1	1.63	0.72	3.53E‐07	2.22	0.028	2.21	0.057	1.00	1
Malate	1	1.61	0.64	1.43E‐05	1.70	0.094	1.61	0.110	1.05	1
N1‐acetylspermine	1	1.58	0.64	1.84E‐05	2.66	0.031	2.02	0.126	1.31	0.900
N‐acetylglutamate	1	1.53	0.60	6.28E‐05	2.86	0.006	2.15	0.044	1.33	0.906
Uridine diphosphate‐N‐acetylglucosamine	3	1.50	0.62	3.13E‐05	2.33	0.060	1.74	0.249	1.34	0.900

*Note:* ID level: Identification level; ID level 1: identified metabolite; ID level 3: tentative structural identification of metabolite.

Abbreviations: Ctrl, Non‐periodontitis controls; FC, Fold Change; Gen, Generalized stage III/IV periodontitis; Loc, Localized stage III/IV periodontitis; VIP, Variable importance in projection score; VIP Pred, Predictive VIP score; ρ, Correlation coefficient.

^**a**^ Top candidate metabolites based on the results shown in Figure 2B and Figure 3.

^**b**^ Taurine and norleucine are listed twice (as ID level 1 and level 3).

**FIGURE 3:**
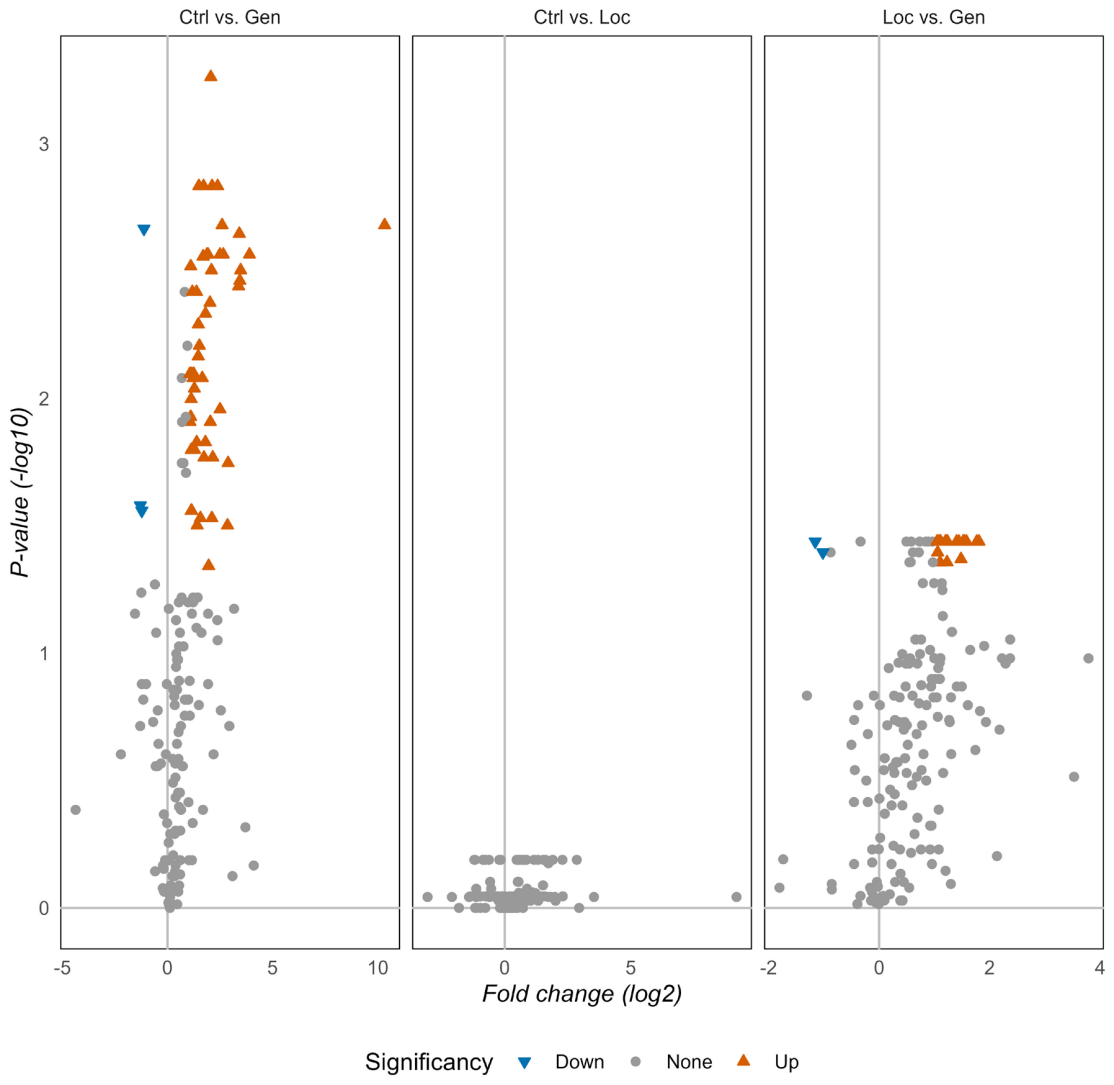
Statistically significant metabolites are color coded (positive fold change or blue negative fold changes) in the volcano plot comparisons (orange = adjusted *p* value < 0.05 and fold change > 2, blue = adjusted *p* value < 0.05 and fold change < 2 or gray = non‐significant) for three group comparisons (Ctrl = control group, Loc = localized periodontitis, Gen = generalized periodontitis). Volcano plots display Log_2_ of the fold change on the *x*‐axis and −Log_10_ of the *p* values on the *y*‐axis. *p* values were computed using Wilcoxon Rank‐Sum (Ctrl: *n* = 16, Loc: *n* = 18, and Gen: *n* = 20).

In volcano plots (Figure 3), we show the results of the pairwise Wilcoxon rank sum tests, revealing significant discrimination for a series of metabolites between patients with generalized stage III/IV periodontitis and controls (*p* < 0.05) as well as between patients with localized stage III/IV periodontitis and generalized stage III/IV periodontitis (*p* < 0.05). There was no significant difference between localized stage III/IV periodontitis patients and controls.

## 4 Discussion

### 4.1 Key Results

Metabolomic fingerprints analyzed from oral rinse samples could distinguish between severe periodontitis (stage III/IV) patients and non‐periodontitis controls. Metabolomic profiles also distinguished between controls vs. generalized stage III/IV periodontitis and between localized stage III/IV periodontitis vs. generalized stage III/IV periodontitis.

Different variables for periodontitis were explored to explain differences in metabolomic profiles between controls and patients with severe periodontitis. In addition to the dichotomization of the extent of periodontitis (localized or generalized), we examined the number of teeth affected with ≥ 33% alveolar bone loss and the number of sites with PPD ≥ 6 mm. The number of sites with PPD of ≥ 6 mm was the best periodontal variable that explained the differences in metabolomic profiles between controls and patients with severe periodontitis; we suggest that the number of sites with deep pockets (PPD ≥ 6 mm) is directly correlated to a higher extent of periodontal inflammation and thus a higher inflammatory burden to the patient. In contrast, radiographic alveolar bone loss does not per se reflect the current activity of periodontal inflammation; rather, it is more regarded as a result of periodontal breakdown in the history of the patient. This corresponds to the results of a larger study (*n* = 909), which also showed associations between salivary metabolites and increasing PPD‐related measures, suggesting that PPD reflects the current disease activity [38].

Remarkably, as visualized in the volcano plots, no significantly different metabolites were found between controls and localized stage III/IV periodontitis. We suspect that the difference in the oral inflammatory burden between these groups is not large enough to find differences in metabolites from oral rinses.

With the current pilot study, we also would like to show the feasibility of obtaining metabolomic fingerprints from oral rinse samples using LC–MS/MS analysis, irrespective of periodontal status. The study showcases the usefulness of applying LC–MS/MS‐based metabolomics for screening and monitoring of severe periodontitis and underlying biochemical patterns. The collection of oral rinses is a simple, non‐invasive, fast sampling technique that is easy to apply and can be scaled up for point‐of‐care, at‐home‐use, and in epidemiological studies. While NMR is a non‐destructive analytical technique for metabolomic markers that provides comprehensive structural information and enables absolute quantification without the need for standards, its relatively lower sensitivity and lengthy analysis time limit its applicability. In contrast, LC–MS/MS offers superior sensitivity, broader metabolite coverage, and high throughput, making it particularly well‐suited for analyzing diluted samples (such as oral rinse samples), untargeted investigations, and non‐invasive sample types, which are often available in large quantities. The current experience acquired will be utilized in large sample experiments for epidemiological purposes. The metabolites currently identified could be considered target metabolites when they are applied to any type of test strips”. The latter would be a cost‐effective method and a major advancement in the screening and monitoring of patients with severe periodontitis.

### 4.2 Metabolomic Signature for Periodontitis

Interestingly, nucleotides were the predominant group among the most significant biomarkers for severe periodontitis. These nucleotides are part of the purine metabolism (deoxyadenosine monophosphate, adenosine diphosphate ribose, inosine monophosphate), pyrimidine metabolism (uridine monophosphate, cytidine monophosphate) or citicoline metabolism, or (citicoline) pathways. Citicoline functions as an intermediate in the membrane phospholipid synthesis, but is also converted into cytidine monophosphate [39, 40]. Increased levels of adenosine diphosphate and cytidine monophosphate in saliva samples of periodontitis patients compared to controls have been reported earlier [41]. For the increased amounts of purine and pyrimidine metabolites in periodontitis patients, it is suggested that the cell metabolism in gingival fibroblasts changes in response to bacterial pathogens, resulting in increased DNA synthesis and mRNA transcription for an acute inflammatory response [42, 43].

The second largest group of metabolites associated with severe periodontitis were (modified) amino acids (taurine, methionine, isoleucine, norleucine, diaminopimelate, O‐acetylserine) and peptides (carnosine, glutathione reduced [GSH]). Indeed, salivary levels of taurine, methionine, and carnosine have been found significantly higher in generalized periodontitis patients compared to controls, regardless of the systemic status of the subjects [44]. Other studies also reported increased salivary levels of taurine [45] and isoleucine [46] in periodontitis patients compared to controls. The literature shows increased [47] and reduced levels of GSH [48] in periodontitis compared to controls. Taurine is the most abundant amino acid present in polymorphonuclear leukocytes and showed to be tissue protective in models of oxidant‐induced injury [49, 50]. Methionine can be converted into methyl mercaptan (methanethiol) by anaerobic, asaccharolytic bacteria and is responsible for oral malodor (a common symptom of periodontitis) and possibly involved in biofilm formation, cytokine response, and decreased collagen synthesis in periodontitis [51, 52]. Isoleucine is part of the branched‐chain amino acids (BCAAs), which may induce the expression of host defense peptides (i.e., β‐defensins) [53, 54]. GSH is the most important redox regulator that controls inflammatory processes. GSH removes reactive oxygen species (ROS) to protect normal cellular function from oxidative damage [55]. Furthermore, taurine, methionine, O‐acetylserine, and GSH are all involved in sulfur metabolism. These are attractive metabolites for pathogens, because sulfur is essential for bacterial growth [56].

Also, dicarboxylic acids (i.e., 3‐dehydroshikimate, methylmalonate, succinate, malate) were found to be associated with severe periodontitis in our study. Other studies found higher levels of succinate [41, 57, 58, 59] and malate [41] in saliva samples of periodontitis patients compared to controls. Contradicting results were found for methylmalonate that was negatively correlated to clinical markers for periodontitis (bleeding on probing, clinical attachment loss, and periodontal pocket depth) [60]. Methylmalonate, also described as methylmalonic acid and involved in the catabolism of pyrimidines, can affect mitochondrial functions and is associated with chronic diseases (e.g., diabetes, obesity, and cardiovascular disease) and oxidative stress [61]. The presence of elevated succinate levels may activate the succinate receptor 1 (SUCNR1) in periodontal tissues, leading to the expression of inflammatory mediators and bone resorption [62].

Finally, the following metabolites are probably of bacterial origin: 3‐dehydroshikimate, N1‐acetylspermine, diaminopimelate [DAP], and uridine diphosphate‐N‐acetylglucosamine [UDP‐GlcNAc]. 3‐Dehydroshikimate is part of the shikimate pathway, which is not found in mammals [63]. N1‐acetylspermine is a polyamine, derived from bacteria. After bacterial cell lysis, polyamines will be released in the oral cavity [64]. Polyamines were shown to affect polymorphonuclear leukocytes by forcing these cells to undergo apoptosis [64, 65]. DAP and UDP‐GlcNAc are reported to be essential building blocks of the peptidoglycan layer of the bacterial cell wall [66, 67].

### 4.3 Strengths and Limitations

Previous studies on metabolomics associated with periodontitis predominantly focused on the use of whole saliva or gingival crevicular fluid [9, 68]. Here we present the first study demonstrating the use of oral rinses for metabolomic markers using LC–MS/MS. Across the various studies, differences in metabolomic profiles may be attributed to differences in instrumental analysis (e.g., LC–MS/MS vs. NMR), cohorts, and sample matrices (such as oral rinse, whole saliva, or GCF). Despite differences as a result of methodological aspects, many of the candidate metabolites associated with severe periodontitis from the oral rinses in our study were also found to be associated with periodontitis in previous metabolomic studies performed with whole saliva or gingival crevicular fluid (see Section 4.2).

One study performing metabolomic profiling of whole saliva to predict periodontal inflammation found that supragingival plaque might influence the detection of metabolites in the subgingival area, which may be a potential limitation of using whole saliva [69]. In our study, we instructed all participants to vigorously rinse through the oral cavity for 30 s. Thereby, we assume to include the gingival crevicular fluid components, which may contain metabolomic markers reflecting periodontal destruction and inflammation. This is further supported by our main finding that the metabolomic signature for severe periodontitis correlates with the number of sites with PPD ≥ 6 mm.

Patient characteristics, such as diabetes mellitus and obesity, which are correlated with periodontitis, were not excluded from our study. In our cohort, patients with self‐reported diabetes were present in localized periodontitis (*n* = 1) and generalized periodontitis (*n* = 3). Interestingly, some metabolomic markers for periodontitis in this study were found to be upregulated in diabetes mellitus, such as isoleucine levels [41]. Therefore, it should be taken into consideration that, due to comorbidity, metabolomic markers from other systemic diseases might also be detected in patients with periodontitis [70]. Also, in the current study, some patients are suspected to have a high risk for cardiovascular disease events, as we reported in our previous larger ‘parent study’ [21]. Therefore, we recommend that future research validates current results across larger and distinct population subgroups, for instance, based on smoking status, BMI, or HbA1c levels. Nevertheless, the current systemic heterogeneity among the individuals of this study can also be considered an advantage, since despite the heterogeneity in the present comorbidities, the metabolomic profile for periodontitis in this study has a very strong top list of candidate metabolites that can serve as a basis for future studies and applications.

Another limitation of the study is the absence of stage I/II periodontitis patients, as such patients are rarely referred to our clinic. Also, the number of controls is unbalanced compared to the total number of subjects suffering from severe periodontitis. On the other hand, we have also analyzed data for 3 groups: control (*n* = 16), localized (*n* = 18), generalized (*n* = 20), and this distribution of individuals per group is slightly less unbalanced. Furthermore, full mouth plaque and bleeding scores were not assessed in our control group. Our control group may therefore encompass both periodontally healthy and gingivitis patients. Nevertheless, the metabolomic profiles were still able to clearly distinguish between severe periodontitis and controls. Future studies should take into consideration above‐mentioned aspects. Currently, our metabolomic signature identified here in this study, could as of yet not be fully generalized, because there might be a selection bias due to the type of periodontitis patients referred to our periodontology department. However, many of our identified metabolomic markers for severe periodontitis have also been identified in previous studies that investigated metabolomic markers in periodontitis patients (see Section 4.2).

Finally, the current analysis is exploratory, aimed at identifying potential biomarkers rather than providing definitive diagnostic conclusions. Further testing and appropriate validation are necessary before these potential biomarkers can be used in a diagnostic setting. Proper validation should include diagnostic performance metrics, such as accuracy, sensitivity, specificity, positive predictive values (PPV), and negative predictive values (NPV), ideally following the STARD guidelines [71].

## 5 Conclusion

We identified potential metabolomic biomarkers and a distinct metabolomic signature for stage III/IV periodontitis that relate to inflammation responses and bacteria. We show that oral rinsing is a valuable and fast alternative to whole saliva sampling for studies into a metabolomic signature of severe periodontitis. Oral rinsing has the additional benefit of allowing sampling of the oral metabolome in case of hyposalivation; however, this specific group of patients was not investigated in the present study, and further research is recommended. Our analysis of oral rinses and the observed wide coverage of metabolites, 175 metabolites detected, was made possible by employing high‐sensitivity mass spectrometry analysis and a dedicated LC–MS/MS‐based workflow. These observations can be a basis for further studies into screening to identify subjects with the risk of having severe periodontitis.

## Author Contributions

**Madeline X. F. Kosho, Bruno G. Loos:** conceptualization. **Madeline X. F. Kosho, Alessio Ciurli, Bruno G. Loos:** methodology. **Madeline X. F. Kosho, Alessio Ciurli, Martin Giera, Jacques J. C. Neefjes, Bruno G. Loos:** formal analysis. **Madeline X. F. Kosho, Alessio Ciurli:** data curation. **Madeline X. F. Kosho, Alessio Ciurli:** investigation. **Madeline X.F. Kosho, Alessio Ciurli:** writing – original draft preparation. **Madeline X. F. Kosho, Alessio Ciurli, Martin Giera, Jacques Neefjes, Bruno G. Loos:** writing – review and editing; **Madeline X. F. Kosho, Alessio Ciurli:** visualization. **Martin Giera, Jacques Neefjes, Bruno G. Loos:** supervision. **Madeline X. F. Kosho:** project administration. **Bruno G. Loos:** funding acquisition.

## Conflicts of Interest

The authors declare no conflicts of interest.

## Supporting information


Data S1.

Data S2.

## Data Availability

The data that support the findings of this study are available from the corresponding author upon reasonable request.
