# Supplementary material for: Metabolomic Profiles of Oral Rinse Samples to Distinguish Severe Periodontitis Patients From Non‐Periodontitis Controls
Source: J Periodontal Res. 2025 Mar 14;60(8):762–74. doi: 10.1111/jre.13379 (PMC12476085; doi:10.1111/jre.13379)
Supplement: Supplementary file 1 — Data S1. [file JRE-60-762-s001.docx]

**Fig. S1**Extent of periodontitis – Partial Least-Squares Discriminant Analysis (PLS-DA) model diagnostic plots.

Top left: barplot displays model parameters (R2Y = explained variance and Q2Y predictive performance). Top right: permutation plot, the R2Y and Q2Y of the model are compared with the corresponding values obtained after random permutation of the *y* variable (extent of periodontitis). Bottom left: observation diagnostic displays score distance (SD) and orthogonal distance (OD). Bottom right: score plot, the number of components and the cumulative R2X, R2Y and Q2Y are indicated below the plot.

**Fig. S2** Number of teeth with alveolar bone loss ≥33% – Partial Least Squares (PLS) model diagnostic plots.

Top left: barplot displays model parameters (R2Y = explained variance and Q2Y predictive performance). Top right: permutation plot, the R2Y and Q2Y of the model are compared with the corresponding values obtained after random permutation of the *y* variable (number of teeth with alveolar bone loss ≥33%). Bottom left: observation diagnostic displays score distance (SD) and orthogonal distance (OD). Bottom right: score plot, the number of components and the cumulative R2X, R2Y and Q2Y are indicated below the plot.

**Fig. S3**Number of sites with Probing Pocket Depth (PPD) ≥6 mm –  Partial Least Squares (PLS) model diagnostic plots.

Top left: barplot displays model parameters (R2Y = explained variance and Q2Y predictive performance). Top right: permutation plot, the R2Y and Q2Y of the model are compared with the corresponding values obtained after random permutation of the *y* variable (number of sites with PPD ≥6 mm). Bottom left: observation diagnostic displays score distance (SD) and orthogonal distance (OD). Bottom right: score plot, the number of components and the cumulative R2X, R2Y and Q2Y are indicated below the plot.

**Fig. S4**Number of sites with Probing Pocket Depth (PPD) ≥6 mm – (Orthogonal-Partial Least Squares) OPLS model diagnostic plots.

Top left: barplot displays model parameters (R2Y = explained variance and Q2Y predictive performance). Top right: permutation plot, the R2Y and Q2Y of the model are compared with the corresponding values obtained after random permutation of the *y* variable (number of sites with PPD ≥6 mm). Bottom left: observation diagnostic displays score distance (SD) and orthogonal distance (OD). Bottom right: score plot, the number of components and the cumulative R2X, R2Y and Q2Y are indicated below the plot.
