# Supplementary material for: Metabolomic Profiles of Oral Rinse Samples to Distinguish Severe Periodontitis Patients From Non‐Periodontitis Controls
Source: J Periodontal Res. 2025 Mar 14;60(8):762–74. doi: 10.1111/jre.13379 (PMC12476085; doi:10.1111/jre.13379)
Supplement: Supplementary file 2 — Data S2. [file JRE-60-762-s002.docx]

**TABLE S1** Mass Spectrometry (MS) settings

| MS Instrument | |
| --- | --- |
| Instrument type | Q-TOF instrument  (TripleTOF 6600, Sciex) |
| Source | |
| Source Type | ESI (Turbo V Ion Source, Sciex) |
| CUR gas | 30 |
| GAS 1 | 50 |
| GAS 2 | 50 |
| ISVF | 5500 V (-4500 V) |
| TEM | 500°C |
| TOF MS | |
| Duration | 13.801 min |
| Cycles | 974 |
| Cycle time | 0.8502 s |
| DP | 80 eV (-80 eV) |
| CE | 10 eV (-10 eV) |
| Start mass | 75 Da |
| End mass | 650 Da |
| Accumulation time | 0.079985 s |
| Product Ion | |
| Acquisition method | IDA |
| With intensity greater than | 100 cps |
| Maximum number of candidates to monitor per cycle | 18 |
| Exclude former target ions | Never |
| Mass tolerance | 50 ppm |
| DP | 80 eV (-80 eV) |
| CE | 30 eV (-30 eV) |
| CES | 15 eV (-15 eV) |
| Start mass | 35 Da |
| End mass | 650 Da |
| Accumulation time | 0.040012 ms |

**TABLE S2** Mass Spectrometry Data Independent AnaLysis (MS-DIAL) settings

| MS-DIAL Version | 4.80 |
| --- | --- |
| Project | |
| MS1 Data type | Profile |
| MS2 Data type | Profile |
| Ion mode | Positive (Negative) |
| Target | Metabolomics |
| Mode | ddMSMS |
| Data collection parameters | |
| Retention time begin | 0.5 |
| Retention time end | 12 |
| Mass range begin | 75 |
| Mass range end | 650 |
| MS2 mass range begin | 30 |
| MS2 mass range end | 650 |
| Centroid parameters | |
| MS1 tolerance | 0.01 |
| MS2 tolerance | 0.025 |
| Isotope recognition and data processing | |
| Maximum charged number | 2 |
| Number of threads | 8 |
| Peak spotting parameters | |
| Mass slice width | 0.1 |
| Peak detection parameters | |
| Smoothing method Linear | LinearWeighted Moving Average |
| Smoothing level | 2 |
| Minimum peak width | 5 |
| Minimum peak height | 500 (300) |
| Deconvolution parameters | |
| Sigma window value | 0.5 |
| MS2Dec amplitude cut off | 1 |
| Exclude after precursor | True |
| Keep isotope until | 0.5 |
| Keep original precursor isotopes | False |
| MSP file and MS/MS identification setting | |
| MSP file | In-house library |
| Retention time tolerance | 0.3 |
| Accurate mass tolerance (MS1) | 0.01 |
| Accurate mass tolerance (MS2) | 0.05 |
| Identification score cut off (%) | 70 |
| Using retention time for scoring | True |
| Using retention time for filtering | True |
| Text file and post identification (retention time and accurate mass based) setting | |
| Retention time tolerance | 0.1 |
| Accurate mass tolerance | 0.01 |
| Identification score cut off | 85 |
| Advanced setting for identification | |
| Relative abundance cut off | 0 |
| Top candidate report | False |
| Adduct ion setting | |
| [M+H]+ ([M-H]-) | True |
| [M+NH4]+ ([M+FA-H]-) | True |
| [M+Na]+ ([M+Cl]-) | True |
| [M+K]+ | True |
| [M+H-H2O]+ ([M-H2O-H]-) | True |
| [M+2H]2+ ([M-2H]2-) | True |
| Alignment parameters setting | |
| Retention time tolerance | 0.3 |
| MS1 tolerance | 0.02 |
| Retention time factor | 0.5 |
| MS1 factor | 0.5 |
| Peak count filter | 0 |
| N% detected in at least one group | 100 |
| Remove feature based on peak height fold-change | True |
| Sample max / blank average | False |
| Sample average / blank average | 2 |
| Keep identified and annotated metabolites | False |
| Keep removable features and assign the tag for checking | False |
| Gap filling by compulsion | True |
| Tracking of isotope labels | |
| Tracking of isotopic labels | False |
| Ion mobility | |
| Ion mobility data | False |

**TABLE S3** Identified metabolites for stage III/IV periodontitis

| **Metabolites** | **ID**  **level** | **VIP** | $\boldsymbol{\rho}$ | **Corrected**  **P-**  **value** | **FC**  **Ctrl.**  **vs.**  **Gen.** | **P-value**  **Ctrl.**  **vs.**  **Gen.** | **FC**  **Loc.**  **vs.**  **Gen.** | **P-value**  **Loc.**  **vs.**  **Gen.** | **FC**  **Ctrl.**  **vs.**  **Loc.** | **P-value**  **Ctrl.**  **vs.**  **Loc.** |
| --- | --- | --- | --- | --- | --- | --- | --- | --- | --- | --- |
| 2-ACETAMIDO-2-DEOXY-BETA-D-GLUCOSYLAMINE | 3 | 2.030795 | 0.771077 | 1.48E-08 | 14.91972 | 0.002717 | 5.162328 | 0.104711 | 2.89011 | 0.647332 |
| DEOXYADENOSINE MONOPHOSPHATE | 1 | 1.966392 | 0.779869 | 7.89E-09 | 7.368296 | 0.017911 | 4.878753 | 0.109743 | 1.51028 | 0.906137 |
| TAURINE | 1 | 1.941253 | 0.723905 | 2.80E-07 | 3.287232 | 0.001464 | 2.296145 | 0.036431 | 1.43163 | 0.647332 |
| ADENOSINE DIPHOSPHATE RIBOSE | 1 | 1.937173 | 0.608839 | 4.99E-05 | 10.4323 | 0.003622 | 4.668379 | 0.104711 | 2.23467 | 0.647332 |
| DIAMINOPIMELATE | 3 | 1.907763 | 0.707727 | 6.73E-07 | 10.66919 | 0.002253 | 3.730377 | 0.09362 | 2.86008 | 0.815483 |
| URIDINE MONOPHOSPHATE | 1 | 1.902858 | 0.72385 | 2.81E-07 | 3.326988 | 0.017032 | 2.137196 | 0.108984 | 1.55671 | 0.906137 |
| 3-DEHYDROSHIKIMATE | 3 | 1.879474 | 0.704789 | 7.84E-07 | 3.805297 | 0.002717 | 2.221017 | 0.071444 | 1.71331 | 0.647332 |
| METHIONINE | 1 | 1.82263 | 0.611319 | 4.56E-05 | 11.14984 | 0.003134 | 5.168427 | 0.088329 | 2.1573 | 0.869292 |
| ISOLEUCINE | 1 | 1.812902 | 0.699037 | 1.05E-06 | 5.644056 | 0.002717 | 3.402962 | 0.036431 | 1.65857 | 0.906137 |
| CITICOLINE | 3 | 1.801843 | 0.748696 | 6.46E-08 | 3.488579 | 0.014831 | 2.085229 | 0.177673 | 1.673 | 0.900355 |
| CARNOSINE | 3 | 1.800625 | 0.71014 | 5.93E-07 | 6.048901 | 0.002085 | 2.988685 | 0.036431 | 2.02393 | 0.93721 |
| TAURINE | 3 | 1.792237 | 0.67465 | 3.40E-06 | 3.501431 | 0.004637 | 2.490878 | 0.082438 | 1.4057 | 0.900355 |
| NORLEUCINE | 1 | 1.781061 | 0.691307 | 1.54E-06 | 4.057809 | 0.004205 | 2.871744 | 0.036431 | 1.41301 | 1 |
| CYTIDINE MONOPHOSPHATE | 3 | 1.764574 | 0.716342 | 4.25E-07 | 3.231923 | 0.002767 | 2.354546 | 0.036431 | 1.37263 | 0.906137 |
| O-ACETYLSERINE | 3 | 1.756209 | 0.763257 | 2.52E-08 | 2.270903 | 0.003811 | 2.133053 | 0.036431 | 1.06463 | 1 |
| INOSINE-MONOPHOSPHATE | 1 | 1.755201 | 0.680578 | 2.58E-06 | 5.647872 | 0.011023 | 1.702673 | 0.287554 | 3.31706 | 0.667831 |
| GLUTATHIONE REDUCED | 1 | 1.752617 | 0.708216 | 6.56E-07 | 3.88204 | 0.045392 | 2.64779 | 0.135104 | 1.46614 | 0.93721 |
| SORBOSE | 1 | 1.744672 | 0.737245 | 1.30E-07 | 2.218825 | 0.007999 | 1.601947 | 0.305573 | 1.38508 | 0.647332 |
| NORLEUCINE | 3 | 1.736722 | 0.682466 | 2.36E-06 | 3.147628 | 0.008304 | 2.313493 | 0.036431 | 1.36055 | 0.949335 |
| METHYLMALONATE | 1 | 1.685886 | 0.697112 | 1.16E-06 | 2.194405 | 0.015892 | 1.848396 | 0.036431 | 1.18719 | 1 |
| SUCCINATE | 1 | 1.633206 | 0.719744 | 3.53E-07 | 2.2187 | 0.027576 | 2.210631 | 0.056502 | 1.00365 | 1 |
| MALATE | 1 | 1.613138 | 0.641452 | 1.43E-05 | 1.695319 | 0.09401 | 1.614102 | 0.109743 | 1.05032 | 1 |
| N1-ACETYLSPERMINE | 1 | 1.580266 | 0.63519 | 1.84E-05 | 2.6601 | 0.031477 | 2.024078 | 0.126213 | 1.31423 | 0.900355 |
| GUANOSINE MONOPHOSPHATE | 1 | 1.57227 | 0.567703 | 0.000201 | 5.218734 | 0.088945 | 1.895439 | 0.475409 | 2.75331 | 0.900355 |
| METHIONINE | 3 | 1.551945 | 0.498327 | 0.001452 | 6.287419 | 0.002717 | 2.059153 | 0.149026 | 3.0534 | 0.647332 |
| PIPECOLATE | 1 | 1.532458 | 0.405836 | 0.011471 | 4.169127 | 0.000546 | 2.912233 | 0.036431 | 1.43159 | 0.78987 |
| N-ACETYLGLUTAMATE | 1 | 1.526407 | 0.602435 | 6.28E-05 | 2.862314 | 0.006202 | 2.147834 | 0.043939 | 1.33265 | 0.906137 |
| URIDINE DIPHOSPHATE-N-ACETYLGLUCOSAMINE | 3 | 1.503585 | 0.621436 | 3.13E-05 | 2.3338 | 0.060382 | 1.743431 | 0.248963 | 1.33862 | 0.900355 |
| NICOTINAMIDE | 1 | 1.48278 | 0.329052 | 0.043673 | 5.220345 | 0.001464 | 1.397847 | 0.109743 | 3.73456 | 0.647332 |
| THIOPURINE S-METHYLETHER | 3 | 1.482548 | -0.34064 | 0.036371 | 0.460415 | 0.002153 | 0.543852 | 0.040169 | 0.84658 | 0.906137 |
| GLYCERALDEHYDE | 1 | 1.467065 | 0.457317 | 0.003894 | 1.369217 | 0.139065 | 1.071992 | 0.25827 | 1.27726 | 1 |
| TYROSINE | 1 | 1.462385 | 0.532008 | 0.000587 | 1.927863 | 0.006202 | 1.95067 | 0.036431 | 0.98831 | 0.900355 |
| S-ADENOSYLHOMOCYSTEINE | 3 | 1.426488 | 0.535985 | 0.000524 | 2.76634 | 0.005112 | 3.497375 | 0.036431 | 0.79098 | 0.900355 |
| EICOSAPENTAENOATE | 1 | 1.426057 | 0.392286 | 0.014852 | 4.313977 | 0.029466 | 2.422896 | 0.186209 | 1.7805 | 0.906137 |
| CYCLIC AMP | 3 | 1.425612 | 0.431457 | 0.006838 | 1.602623 | 0.017911 | 1.218947 | 0.182851 | 1.31476 | 0.900355 |
| GLUTAMATE | 3 | 1.377424 | 0.504342 | 0.001244 | 2.153607 | 0.003023 | 1.461453 | 0.043939 | 1.47361 | 0.647332 |
| PHENYLALANINE | 1 | 1.36169 | 0.450203 | 0.004566 | 1.840696 | 0.019568 | 1.584132 | 0.088329 | 1.16196 | 1 |
| OPHTHALMATE | 3 | 1.333405 | 0.371381 | 0.021695 | 4.351443 | 0.001464 | 2.098386 | 0.114399 | 2.07371 | 0.876003 |
| UROCANATE | 1 | 1.32036 | 0.402745 | 0.012178 | 2.181303 | 0.010039 | 1.57503 | 0.088329 | 1.38493 | 0.900355 |
| SUBERATE | 1 | 1.314779 | -0.37546 | 0.020187 | 0.688286 | 0.083081 | 0.768318 | 0.15995 | 0.89583 | 1 |
| 2-HYDROXYBUTYRATE | 1 | 1.314332 | 0.351207 | 0.030613 | 1.592964 | 0.060382 | 1.284443 | 0.186209 | 1.2402 | 0.93721 |
| DEOXYGUANOSINE-MONOPHOSPHATE | 1 | 1.313435 | 0.468449 | 0.003015 | 5.796479 | 0.167694 | 1.938773 | 0.67274 | 2.98977 | 0.900355 |
| CYTOSINE | 3 | 1.301369 | 0.459521 | 0.003704 | 2.618508 | 0.014831 | 1.932411 | 0.126213 | 1.35505 | 0.900355 |
| DEOXYCARNITINE | 1 | 1.292554 | 0.546562 | 0.000385 | 2.187509 | 0.027576 | 1.994834 | 0.104588 | 1.09659 | 0.917931 |
| OXOGLUTARATE | 1 | 1.247363 | 0.368976 | 0.022629 | 1.693472 | 0.017911 | 1.644623 | 0.040169 | 1.0297 | 0.906137 |
| O-ACETYLCARNITINE | 3 | 1.236283 | 0.402218 | 0.012302 | 2.600839 | 0.07949 | 2.157578 | 0.104588 | 1.20544 | 1 |
| SEROTONIN | 3 | 1.212105 | 0.294327 | 0.072859 | 1283.774 | 0.002085 | 2.19451 | 0.053087 | 584.994 | 0.906137 |
| OXOPROLINE | 1 | 1.185343 | 0.358981 | 0.026874 | 1.829988 | 0.011796 | 1.406838 | 0.036431 | 1.30078 | 1 |
| XANTHINE | 3 | 1.177204 | 0.310838 | 0.057502 | 3.681653 | 0.002717 | 1.962286 | 0.043939 | 1.87621 | 0.900355 |
| GLUTARATE | 1 | 1.159403 | 0.324619 | 0.046761 | 4.256047 | 0.003134 | 1.71223 | 0.191796 | 2.48567 | 0.647332 |
| PYRIDOXAMINE | 3 | 1.143096 | 0.317915 | 0.051764 | 3.258107 | 0.002767 | 2.869903 | 0.036431 | 1.13527 | 0.906137 |
| N,N,N-TRIMETHYLLYSINE | 3 | 1.138498 | 0.437279 | 0.006046 | 2.317754 | 0.008304 | 1.989532 | 0.053087 | 1.16497 | 0.906137 |
| HYPOXANTHINE | 1 | 1.136843 | 0.333024 | 0.041047 | 2.151087 | 0.011796 | 1.292404 | 0.149026 | 1.66441 | 0.900355 |
| CORTISONE | 1 | 1.129442 | -0.1914 | 0.249667 | 0.661109 | 0.053674 | 0.728897 | 0.383986 | 0.907 | 0.900355 |
| ADENOSINE-MONOPHOSPHATE | 1 | 1.112435 | 0.324957 | 0.04652 | 4.564743 | 0.249345 | 1.361868 | 0.791379 | 3.35182 | 0.906137 |
| TRYPTOPHAN | 1 | 1.105352 | 0.366275 | 0.023717 | 2.70329 | 0.060382 | 1.696169 | 0.133479 | 1.59376 | 0.906137 |
| DEOXYADENOSINE MONOPHOSPHATE | 3 | 1.094694 | 0.401054 | 0.012581 | 5.173005 | 0.074124 | 13.80922 | 0.104711 | 0.37461 | 0.906137 |
| GLYCOCHENODEOXYCHOLATE | 1 | 1.071379 | 0.296875 | 0.070299 | 4.451386 | 0.017032 | 0.913433 | 0.865353 | 4.87325 | 0.647332 |
| NORVALINE | 3 | 1.067546 | 0.401778 | 0.012407 | 2.092045 | 0.007999 | 1.729983 | 0.053087 | 1.20929 | 1 |
| HISTIDINE | 1 | 1.065229 | 0.232236 | 0.160604 | 2.096325 | 0.012347 | 2.162657 | 0.036431 | 0.96933 | 0.969345 |
| OROTATE | 1 | 1.047416 | 0.161953 | 0.331336 | 2.812336 | 0.001464 | 0.792018 | 0.036431 | 3.55085 | 0.906137 |
| 3,4 DIHYDROXYMANDELATE | 3 | 1.043016 | 0.334914 | 0.039844 | 3.217009 | 0.411978 | 3.541853 | 0.16864 | 0.90828 | 0.906137 |
| INOSINE | 1 | 1.037101 | 0.345394 | 0.03368 | 2.424151 | 0.009135 | 1.519461 | 0.146745 | 1.5954 | 0.900355 |
| CORTISOL 21-ACETATE | 1 | 1.030727 | -0.29109 | 0.076214 | 0.429088 | 0.132184 | 0.851119 | 0.316166 | 0.50415 | 0.900355 |
| CORTISOL 21-ACETATE | 3 | 1.018782 | -0.19866 | 0.231822 | 0.342624 | 0.069961 | 0.790971 | 0.897198 | 0.43317 | 0.647332 |
| N-ACETYLMETHIONINE | 3 | 1.015783 | 0.287344 | 0.080249 | 2.323277 | 0.06311 | 1.410929 | 0.295194 | 1.64663 | 0.647332 |
| 2-HYDROXY-4-(METHYLTHIO)BUTANOATE | 3 | 0.970666 | 0.203553 | 0.220292 | 2.080896 | 0.175895 | 0.892106 | 0.832992 | 2.33257 | 0.876003 |
| ELAIDATE | 3 | 0.965878 | 0.386761 | 0.016454 | 1.507626 | 0.352705 | 1.599544 | 0.207624 | 0.94253 | 0.906137 |
| URATE | 1 | 0.944982 | 0.199121 | 0.230719 | 1.600006 | 0.008304 | 1.495165 | 0.036431 | 1.07012 | 0.906137 |
| AZELATE | 1 | 0.920153 | -0.29547 | 0.0717 | 0.880541 | 0.702056 | 0.730791 | 0.182851 | 1.20491 | 0.900355 |
| RAFFINOSE | 1 | 0.908683 | 0.670297 | 4.15E-06 | 0.665471 | 0.718086 | 1.457936 | 0.832992 | 0.45645 | 0.906137 |
| CYTIDINE 2',3'-CYCLIC PHOSPHATE | 3 | 0.906874 | 0.162042 | 0.331067 | 2.37375 | 0.007999 | 2.067527 | 0.036431 | 1.14811 | 1 |
| DEOXYGUANOSINE | 1 | 0.902498 | 0.207495 | 0.211295 | 7.235632 | 0.031477 | 4.523087 | 0.199595 | 1.59971 | 0.906137 |
| CYTIDINE | 3 | 0.88234 | 0.099542 | 0.55211 | 2.606027 | 0.003811 | 2.717365 | 0.036431 | 0.95903 | 1 |
| XANTHINE | 1 | 0.8787 | 0.195686 | 0.239027 | 2.441073 | 0.015892 | 1.484225 | 0.043891 | 1.64468 | 1 |
| METHYLTHIOADENOSINE | 3 | 0.86879 | 0.253374 | 0.124809 | 3.811904 | 0.132184 | 2.457939 | 0.805364 | 1.55085 | 0.647332 |
| DEOXYADENOSINE | 1 | 0.86144 | 0.190738 | 0.251348 | 3.808921 | 0.069961 | 3.131466 | 0.097067 | 1.21634 | 1 |
| O-ACETYLCARNITINE | 1 | 0.860874 | 0.247475 | 0.134135 | 1.328404 | 0.113106 | 1.336904 | 0.100722 | 0.99364 | 1 |
| TYRAMINE | 1 | 0.838789 | 0.021074 | 0.90006 | 10.85758 | 0.003443 | 0.934929 | 0.146745 | 11.6133 | 0.906137 |
| OXOPROLINE | 3 | 0.816542 | 0.249063 | 0.131575 | 1.367845 | 0.106033 | 1.127924 | 0.114399 | 1.21271 | 1 |
| THYMIDINE | 1 | 0.816024 | 0.18184 | 0.274556 | 4.107685 | 0.012347 | 2.461036 | 0.149026 | 1.66909 | 0.906137 |
| N-ACETYLSERINE | 3 | 0.803994 | 0.171411 | 0.303485 | 1.764199 | 0.003811 | 1.527018 | 0.040169 | 1.15532 | 1 |
| URIDINE | 1 | 0.795841 | 0.323181 | 0.0478 | 1.767541 | 0.175895 | 1.812543 | 0.15995 | 0.97517 | 1 |
| GUANINE | 1 | 0.777325 | 0.305748 | 0.061933 | 1.436519 | 0.260005 | 1.383584 | 0.25827 | 1.03826 | 1 |
| CITRAMALATE | 1 | 0.720371 | 0.388009 | 0.01608 | 1.423412 | 0.352705 | 1.977331 | 0.149026 | 0.71987 | 0.900355 |
| PHOSPHORYLCHOLINE | 3 | 0.714799 | 0.328178 | 0.044268 | 1.460801 | 0.09401 | 1.374397 | 0.186209 | 1.06287 | 0.906137 |
| NICOTINATE | 1 | 0.713749 | 0.079507 | 0.635149 | 2.963838 | 0.029466 | 1.186389 | 0.280091 | 2.4982 | 0.93721 |
| CREATININE | 1 | 0.704009 | 0.222087 | 0.180209 | 1.613899 | 0.012347 | 1.662551 | 0.036431 | 0.97074 | 0.93721 |
| SORBITOL | 1 | 0.703487 | 0.253463 | 0.124673 | 7.678228 | 0.193136 | 1.559309 | 0.51298 | 4.92412 | 0.900355 |
| GUANOSINE | 3 | 0.689197 | 0.200006 | 0.228611 | 1.453158 | 0.06311 | 1.474204 | 0.104711 | 0.98572 | 1 |
| CITRATE | 1 | 0.684823 | 0.216739 | 0.191201 | 1.263752 | 0.512409 | 0.995173 | 0.965442 | 1.26988 | 0.93721 |
| NORMETANEPHRINE | 3 | 0.680566 | 0.031773 | 0.849807 | 2.759823 | 0.006825 | 1.894374 | 0.097067 | 1.45685 | 0.900355 |
| GLUCOSE 1-PHOSPHATE | 3 | 0.677756 | 0.148202 | 0.374553 | 1.189003 | 0.862764 | 0.300159 | 0.644446 | 3.96125 | 0.906137 |
| TRANS-ACONITATE | 3 | 0.646819 | 0.313754 | 0.055079 | 1.366856 | 0.22688 | 1.362678 | 0.199595 | 1.00307 | 1 |
| 4-HYDROXY-L-PHENYLGLYCINE | 3 | 0.643457 | 0.186024 | 0.263474 | 2.033427 | 0.649487 | 3.341135 | 0.239886 | 0.6086 | 0.900355 |
| UREIDOPROPIONATE | 1 | 0.634001 | 0.180634 | 0.277806 | 1.419483 | 0.106033 | 1.796378 | 0.036431 | 0.79019 | 0.906137 |
| ADENOSINE | 1 | 0.615151 | -0.14308 | 0.391456 | 0.80688 | 0.270942 | 0.8673 | 0.383986 | 0.93033 | 1 |
| L-ARGININE | 1 | 0.612686 | 0.342909 | 0.035066 | 1.218011 | 0.139065 | 2.083362 | 0.040169 | 0.58464 | 1 |
| N,N-DIMETHYLARGININE | 1 | 0.596093 | 0.26479 | 0.108148 | 1.247855 | 0.147704 | 1.275927 | 0.108984 | 0.978 | 1 |
| HIPPURATE | 1 | 0.596059 | 0.071639 | 0.669081 | 0.421932 | 0.057814 | 0.958401 | 0.953329 | 0.44025 | 0.647332 |
| N-ACETYLMETHIONINE | 1 | 0.588014 | 0.222537 | 0.179305 | 1.464979 | 0.401198 | 1.142092 | 0.884035 | 1.28272 | 0.917931 |
| MYRISTATE | 1 | 0.580968 | 0.277291 | 0.091899 | 1.084729 | 1 | 1.166939 | 0.67274 | 0.92955 | 0.900355 |
| ALLANTOIN | 1 | 0.576298 | -0.24047 | 0.145878 | 0.860507 | 0.681564 | 1.297958 | 0.58943 | 0.66297 | 0.900355 |
| N-ACETYLGLUCOSAMINE | 1 | 0.569045 | 0.132982 | 0.42608 | 1.997354 | 0.384646 | 3.040453 | 0.15995 | 0.65693 | 0.906137 |
| GLUCOSAMINE 6-PHOSPHATE | 3 | 0.562372 | 0.085185 | 0.611103 | 2.809099 | 0.159772 | 2.405779 | 0.182851 | 1.16765 | 1 |
| N-ACETYLASPARTATE | 1 | 0.558262 | 0.045829 | 0.784688 | 1.466846 | 0.128327 | 1.66882 | 0.100722 | 0.87897 | 1 |
| N-ACETYLNEURAMINATE | 3 | 0.551454 | 0.084748 | 0.61294 | 1.772343 | 0.152125 | 1.916828 | 0.135104 | 0.92462 | 1 |
| N-ACETYLGLUCOSAMINE | 3 | 0.548551 | 0.129447 | 0.438589 | 2.291979 | 0.464304 | 3.81535 | 0.186209 | 0.60073 | 0.900355 |
| ADENINE | 1 | 0.524706 | 0.309483 | 0.058655 | 1.292264 | 0.270942 | 1.456813 | 0.109743 | 0.88705 | 1 |
| HISTAMINE | 3 | 0.522524 | 0.007953 | 0.962204 | 1.987839 | 0.152125 | 1.206273 | 0.146745 | 1.64792 | 1 |
| N-ACETYLPUTRESCINE | 1 | 0.514231 | 0.294968 | 0.072208 | 1.284352 | 0.718086 | 2.81835 | 0.135104 | 0.45571 | 0.840721 |
| 2,3-DIHYDROXYBENZOATE | 1 | 0.511873 | -0.09975 | 0.551271 | 0.428702 | 0.027576 | 0.730311 | 0.67274 | 0.58701 | 0.647332 |
| THEOPHYLLINE | 1 | 0.506019 | -0.28561 | 0.082169 | 1.029645 | 0.8526 | 0.706013 | 0.228775 | 1.45839 | 0.78987 |
| 5-AMINOPENTANOATE | 1 | 0.500568 | 0.172529 | 0.300294 | 1.629461 | 0.277675 | 2.341611 | 0.043891 | 0.69587 | 0.900355 |
| MELIBIOSE | 1 | 0.490922 | -0.00836 | 0.960282 | 0.409198 | 0.026273 | 0.448538 | 0.036431 | 0.91229 | 1 |
| ANILINE-2-SULFONATE | 3 | 0.489828 | -0.12618 | 0.45034 | 1.038553 | 0.554148 | 0.916678 | 0.663102 | 1.13295 | 1 |
| P-HYDROXYPHENYLACETATE | 1 | 0.473429 | -0.24401 | 0.139848 | 1.047749 | 0.967933 | 1.491812 | 0.607595 | 0.70233 | 0.900355 |
| SALICYLATE | 1 | 0.463364 | 0.035758 | 0.831225 | 0.450431 | 0.152125 | 1.926811 | 0.475409 | 0.23377 | 0.906137 |
| D-ALANINE | 1 | 0.453872 | 0.19948 | 0.229863 | 1.337434 | 0.100462 | 1.236295 | 0.267806 | 1.08181 | 0.93721 |
| SORBITOL | 3 | 0.453688 | 0.26222 | 0.111743 | 8.519388 | 0.749967 | 2.110803 | 0.411233 | 4.03609 | 0.93721 |
| GALACTITOL | 1 | 0.451015 | 0.242162 | 0.142969 | 13.04015 | 0.482428 | 1.688675 | 0.58943 | 7.72212 | 1 |
| CYTIDINE 2',3'-CYCLIC PHOSPHATE | 1 | 0.423284 | 0.058749 | 0.726069 | 1.507717 | 0.083081 | 1.413932 | 0.191796 | 1.06633 | 0.906137 |
| CREATINE | 1 | 0.414296 | 0.169546 | 0.308854 | 1.326019 | 0.074124 | 1.210209 | 0.295194 | 1.09569 | 0.981228 |
| N-ACETYLASPARTATE | 3 | 0.407356 | 0.260575 | 0.114091 | 1.188156 | 0.260005 | 1.261637 | 0.267806 | 0.94176 | 1 |
| CYCLIC GMP | 1 | 0.402061 | -0.0302 | 0.857182 | 1.981407 | 0.06311 | 1.338554 | 0.395941 | 1.48026 | 0.871522 |
| 5'-DEOXYADENOSINE | 3 | 0.40084 | 0.088517 | 0.597176 | 1.27051 | 0.159772 | 1.107102 | 0.191796 | 1.1476 | 1 |
| INDOLE-3-ACETATE | 1 | 0.397553 | -0.16133 | 0.333217 | 1.17545 | 0.749967 | 2.299395 | 0.715015 | 0.5112 | 1 |
| MELIBIOSE | 3 | 0.379637 | 0.023569 | 0.8883 | 0.215469 | 0.249345 | 0.55261 | 0.846608 | 0.38991 | 0.900355 |
| METHYGLUTARATE | 1 | 0.379623 | -0.09253 | 0.580582 | 0.870352 | 0.681564 | 0.868016 | 0.207624 | 1.00269 | 0.93721 |
| CYCLIC AMP | 1 | 0.377559 | -0.21235 | 0.200576 | 1.378127 | 0.967933 | 1.072718 | 0.426834 | 1.28471 | 0.900355 |
| ANSERINE | 3 | 0.372232 | 0.097908 | 0.558687 | 1.042839 | 0.066903 | 1.895711 | 0.58943 | 0.5501 | 0.647332 |
| NICOTINATE | 3 | 0.350207 | -0.0125 | 0.940627 | 1.840129 | 0.152125 | 1.003548 | 0.372214 | 1.83362 | 0.93721 |
| MANNOSE | 1 | 0.33605 | -0.0173 | 0.917888 | 1.524355 | 0.497346 | 1.616872 | 0.442736 | 0.94278 | 0.981228 |
| 5-METHYLCYTOSINE | 3 | 0.330921 | -0.11516 | 0.491167 | 1.518562 | 0.738679 | 2.111901 | 0.58943 | 0.71905 | 0.900355 |
| L-ORNITHINE | 3 | 0.328566 | 0.3187 | 0.051157 | 0.976267 | 0.132184 | 2.119325 | 0.036431 | 0.46065 | 1 |
| N-ACETYLPHENYLALANINE | 1 | 0.326507 | -0.12363 | 0.459596 | 1.269126 | 0.749967 | 1.304644 | 0.935511 | 0.97278 | 0.949335 |
| INDOXYL SULFATE | 1 | 0.324197 | -0.06381 | 0.703512 | 0.622656 | 0.186066 | 0.906994 | 0.846608 | 0.6865 | 0.840721 |
| HEPTADECANOATE | 1 | 0.316635 | 0.062121 | 0.711006 | 1.305206 | 0.681564 | 1.222553 | 0.791379 | 1.06761 | 1 |
| QUINATE | 1 | 0.313427 | -0.07727 | 0.644719 | 8.971961 | 0.066903 | 4.381777 | 0.625938 | 2.04756 | 0.871522 |
| THEOBROMINE | 1 | 0.30523 | -0.2763 | 0.093119 | 0.892807 | 0.862764 | 0.493911 | 0.040169 | 1.80763 | 0.647332 |
| SPHINGANINE | 1 | 0.304846 | 0.033737 | 0.840637 | 0.987476 | 0.464304 | 1.148656 | 0.343515 | 0.85968 | 0.647332 |
| 4-HYDROXYBENZOATE | 1 | 0.304452 | -0.05468 | 0.744371 | 2.073444 | 0.128327 | 0.286521 | 0.832992 | 7.23661 | 0.647332 |
| HYDROXYPHENYLLACTATE | 1 | 0.298583 | -0.05466 | 0.744451 | 1.307556 | 0.368479 | 0.971679 | 0.791379 | 1.34567 | 0.969345 |
| TRIGONELLINE | 1 | 0.297014 | -0.05394 | 0.747733 | 3.062382 | 0.083081 | 2.468297 | 0.248963 | 1.24069 | 1 |
| L-CARNITINE | 1 | 0.2896 | 0.117578 | 0.482036 | 1.028383 | 0.953726 | 0.924484 | 0.935511 | 1.11239 | 1 |
| CITRULLINE | 3 | 0.280714 | 0.291517 | 0.075767 | 0.948887 | 0.249345 | 2.139149 | 0.126213 | 0.44358 | 1 |
| N-ACETYLNEURAMINATE | 1 | 0.278299 | -0.03518 | 0.833895 | 1.56112 | 0.411978 | 1.510014 | 0.32967 | 1.03384 | 1 |
| URACIL | 1 | 0.253081 | 0.093291 | 0.577465 | 1.654683 | 0.277675 | 1.168527 | 0.395941 | 1.41604 | 1 |
| 3-METHOXYTYRAMINE | 3 | 0.249228 | 0.01806 | 0.914296 | 0.744081 | 0.22688 | 0.90342 | 0.935511 | 0.82363 | 0.900355 |
| N-ACETYLSEROTONIN | 3 | 0.24707 | 0.149978 | 0.368791 | 0.720739 | 0.277675 | 1.306673 | 0.734018 | 0.55158 | 0.647332 |
| KETOLEUCINE | 3 | 0.242834 | -0.09454 | 0.572344 | 1.044551 | 0.649487 | 0.984161 | 0.824457 | 1.06136 | 1 |
| 3-HYDROXYANTHRANILATE | 3 | 0.221377 | -0.08665 | 0.604965 | 1.1063 | 0.816521 | 0.995884 | 0.588142 | 1.11087 | 0.906137 |
| PANTOTHENATE | 3 | 0.211982 | -0.12003 | 0.472872 | 0.855373 | 0.837149 | 0.761286 | 0.965442 | 1.12359 | 1 |
| 4-GUANIDINOBUTANOATE | 3 | 0.197414 | 0.354134 | 0.029158 | 0.406542 | 0.193136 | 1.431836 | 0.228775 | 0.28393 | 1 |
| CAFFEINE | 1 | 0.194423 | -0.12841 | 0.442305 | 2.24106 | 0.649487 | 1.214635 | 0.357698 | 1.84505 | 0.840721 |
| N-ACETYLGALACTOSAMINE | 3 | 0.193181 | -0.05068 | 0.762532 | 1.522893 | 0.649487 | 1.805132 | 0.316166 | 0.84365 | 0.93721 |
| EPINEPHRINE | 3 | 0.170452 | -0.20551 | 0.215784 | 1.087824 | 0.908291 | 1.068713 | 0.933935 | 1.01788 | 1 |
| N-ACETYLGALACTOSAMINE | 1 | 0.168428 | -0.01377 | 0.93461 | 1.430262 | 0.862764 | 2.231023 | 0.295194 | 0.64108 | 0.900355 |
| N-ACETYLLEUCINE | 1 | 0.15509 | -0.04801 | 0.774689 | 2.23558 | 0.069961 | 1.649429 | 0.157282 | 1.35537 | 1 |
| 4-AMINOBENZOATE | 3 | 0.151131 | -0.12333 | 0.460708 | 1.465352 | 0.816521 | 1.341437 | 0.935511 | 1.09237 | 1 |
| PANTOTHENATE | 1 | 0.147592 | -0.09392 | 0.574902 | 0.92117 | 0.649487 | 0.974278 | 0.915623 | 0.94549 | 0.906137 |
| 5-HYDROXYTRYPTOPHAN | 3 | 0.146695 | 0.108862 | 0.515311 | 1.095877 | 0.512409 | 1.692548 | 0.088329 | 0.64747 | 0.647332 |
| 3-SULFINOALANINE | 3 | 0.146486 | -0.18156 | 0.275316 | 1.554639 | 0.193136 | 2.641759 | 0.036431 | 0.58849 | 0.900355 |
| RIBOFLAVIN | 1 | 0.139511 | 0.012381 | 0.941192 | 0.944241 | 0.8526 | 1.053281 | 0.897198 | 0.89648 | 0.949335 |
| 1-METHYL-L-HISTIDINE | 3 | 0.13859 | -0.10192 | 0.542623 | 1.207289 | 0.621704 | 1.060336 | 0.287554 | 1.13859 | 0.900355 |
| SPERMINE | 1 | 0.130641 | -0.00458 | 0.978228 | 0.725873 | 0.167694 | 0.73676 | 0.287554 | 0.98522 | 1 |
| PROLINE | 1 | 0.128652 | -0.15463 | 0.353959 | 1.440112 | 0.203968 | 2.790586 | 0.042682 | 0.51606 | 0.900355 |
| N-ALPHA-ACETYLLYSINE | 3 | 0.120022 | -0.08746 | 0.601582 | 0.493651 | 0.132184 | 0.551943 | 0.805364 | 0.89439 | 0.647332 |
| 4-PYRIDOXATE | 3 | 0.111773 | -0.04622 | 0.78288 | 1.300162 | 0.307791 | 1.008723 | 0.15995 | 1.28892 | 0.900355 |
| AMINOADIPATE | 3 | 0.087388 | -0.1066 | 0.524131 | 1.303414 | 0.497346 | 1.393682 | 0.135104 | 0.93523 | 0.917931 |
| 1-METHYLADENOSINE | 3 | 0.076071 | 0.178187 | 0.284474 | 0.677923 | 0.277675 | 1.013236 | 0.530352 | 0.66907 | 0.78987 |
| GUANOSINE | 1 | 0.042194 | 0.00756 | 0.964068 | 1.200967 | 0.322356 | 1.197514 | 0.569975 | 1.00288 | 0.981228 |
| TREHALOSE | 1 | 0.040306 | 0.128151 | 0.443226 | 0.048382 | 0.411978 | 0.404339 | 0.146745 | 0.11966 | 0.906137 |
| FERULATE | 3 | 0.019355 | -0.10092 | 0.546588 | 17.14425 | 0.681564 | 11.51878 | 0.305573 | 1.48837 | 0.900355 |
| N-FORMYL-L-METHIONINE | 3 | 0.004894 | 0.066852 | 0.690053 | 0.889234 | 0.429068 | 0.916053 | 0.58943 | 0.97072 | 1 |

ID level: Identification level

ID level 1: identified metabolite; ID level 3: tentative structural identification of metabolite

VIP score: Variable importance in projection score

$\rho$: Correlation coefficient

FC: Fold Change

Ctrl.: Non-periodontitis controls

Loc.: Localized stage III/IV periodontitis

Gen.: Generalized stage III/IV periodontitis
